# Identification and fungicide sensitivity of *Monosporascus lespedezae* sp. nov. causing root rot of *Lespedeza davurica* in Gansu, China

**DOI:** 10.3389/fpls.2026.1869588

**Published:** 2026-06-19

**Authors:** Tong Tong Wang, Yue Yang Zhang, Yan Feng Jia, Jin Qiu Wang, Cheng Yue Hu, Zi Xuan Zhang, Yan Zhong Li

**Affiliations:** 1 State Key Laboratory of Herbage Improvement and Grassland Agro-Ecosystems, College of Pastoral Agriculture Science and Technology, Lanzhou University, Lanzhou, China; 2 Engineering Research Center of Grassland Industry, Ministry of Education, Lanzhou, China

**Keywords:** chemical control, fungal pathogen, Leguminosae, natural grassland, novel species

## Abstract

*Lespedeza davurica* is an important leguminous forage species widely distributed in China’s natural grasslands, where it contributes significantly to ecological stability and sustainable animal husbandry. However, a severe root rot disease, with disease incidence up to 100% has been observed in surveyed areas of Huan County, Gansu Province, China, causing substantial damage to the host plant, while the causal agent remains uncharacterized. Four fungal isolates recovered from symptomatic roots were identified as a novel species of *Monosporascus* based on morphological characteristics and phylogenetic analyses of ITS, LSU, and *Tub2* sequences. The isolates were formally described as *Monosporascus lespedezae* sp. nov. Pathogenicity tests confirmed high virulence, with inoculated plants showing 100% disease incidence and significant reductions in root length (81.3%) and plant height (63.0%) compared to non-inoculated controls (*P* < 0.001). *In vitro* fungicide sensitivity assays showed differential responses among tested compounds: diniconazole exhibited the lowest EC_50_ value (0.5198 μg/mL), whereas carbendazim completely inhibited mycelial growth at 10 μg/mL (EC_50_ = 2.791 μg/mL). Both fungicides show promise for further valuation. This study represents the first report of *M. lespedezae* sp. nov. as a pathogen of *L. davurica*, establishes its taxonomic position, and provides baseline *in vitro* fungicide sensitivity data, thereby laying a scientific foundation for developing effective disease management strategies.

## 1 Introduction

*Lespedeza davurica* is a deciduous shrub in the family Fabaceae, widely distributed across northern, central, and eastern China, thriving in diverse habitats (https://species.sciencereading.cn/home), and is one of the dominant species in the semiarid natural grasslands in China (China species library). *L. davurica* performs a broad array of ecological, nutritional, and medicinal functions. Ecologically, it exhibits strong drought tolerance in semiarid grasslands through several adaptive strategies, including an increased root-to-shoot ratio (Duan et al., 2022), sustained photosynthetic activity, enhanced antioxidant capacity, and rapid recovery after rehydration (Li et al., 2025). These characteristics highlight its critical role in promoting ecosystem stability and enhancing soil quality. Nutritionally, it serves as a high-quality leguminous forage, noted for its adaptability and resistance to heavy and frequent defoliation (Cheng et al., 2004; Wang, 2003). In traditional Chinese medicine, it has been historically utilized to treat various ailments (He, 1993). Recent studies have demonstrated that its extracts can modulate blood glucose levels in Type 1 diabetes (Sharma and Rhyu, 2015), highlighting its potential as a source of novel therapeutic agents.

According to records from the U.S. National Fungus Collections (Farr et al., 2026), 407 disease records are associated with *Lespedeza* species across 12 countries or regions worldwide, involving 79 fungal genera, with most pathogens affecting leaf tissues. Predominant genera include *Phyllachora* spp (Cannon, 1991), *Erysiphe* spp. (https://genebank.rda.go.kr/english/plntDissInfoMain.do), and *Uromyces* spp (Tai, 1979; National Institute of Agricultural Sciences, The Korea Society of Plant Pathology, 2025). Among these, *Uromyces* accounts for over 25% of reported records and is particularly prevalent in several east Asian countries. For the host plant in the current study, *L. davurica*, only two pathogenic fungi have been previously documented: *Uromyces lespedezae-procumbentis* (Tai, 1979) and *Oidium* sp (Zheng and Yu, 1987).

The genus *Monosporascus* (*Diatrypaceae*, *Xylariales*, *Sordariomycetes*) currently comprises 14 recognized species, according to the Index Fungorum database (https://www.indexfungorum.org). Among these, *M. cannonballus* and *M. eutypoides* are prominent pathogens causing root rot and vine decline syndrome in Cucurbitaceae crops, such as *Citrullus lanatus* and *Cucumis melo*, with reports from multiple regions including Korea (https://genebank.rda.go.kr/english/plntDissInfoMain.do), Mexico (Gaytan-Mascorro et al., 2012), and China (Yan et al., 2016). In addition to these pathogenic species, nine others, including *M. bulgaricus*, *M. europaeus* (Crous et al., 2024a), *M. brasiliensis*, *M. caatinguensis*, *M. mossoroensis*, *M. nordestinus*, *M. semiaridus* (Negreiros et al., 2019), *M. ibericus* (Collado et al., 2002), and *M. solitarius* (Crous et al., 2024b) have been functioned as endophytes in host plants. Conversely, *M. auratispora* is the only species not associated with plants, having been isolated from Spanish coastal lagoons (Barnés-Guirado et al., 2025). In contrast, *M. adenantherae* and *M. monosporus* remain poorly characterized owing to the lack of accessible reference isolates or sequence data, with their ecological roles undetermined.

In this study, we identified and characterized a novel species of *Monosporascus* associated with root rot in *L. davurica* through morphological characterization and multigene phylogenetic analyses, which allowed to establish its taxonomic position within the genus. Pathogenicity under controlled conditions and *in vitro* sensitivity to six commonly used fungicides were evaluated. These findings provide essential baseline data for disease management and contribute to a deeper understanding of pathogenic diversity within *Monosporascus*.

## 2 Materials and methods

### 2.1 Sample collection and pathogen isolation

In August 2023, *L. davurica* plants showing root disease symptoms were observed in a natural grassland in Huan County (37°7′N, 116°49′E; altitude 1538.4 m), Gansu Province, China. The site is located on the semiarid Loess Plateau, with a mean annual temperature (MAT) of 7.5 °C, mean annual precipitation of 350 mm, and an average annual sunshine duration of 2596 h. The soil is silty loam with alkaline properties. At this site, five 1 m × 1 m quadrats were randomly established with a minimum spacing of 100 m between quadrats to ensure spatial independence. The plant density at the site was 13 plants m^-^². A total of 20 symptomatic plants were excavated and transported to the laboratory. Root systems were transversely sectioned to inspect the stele. Plants were categorized as having a healthy (white) stele or showing brown discoloration in the stele. Disease incidence (1) was calculated using the following Equation 1:

(1) $$disease\;incidence(\%)=\frac{number\;of\;diseased\;plants}{total\;number\;of\;plants\;examined}\times 100$$

Five representative plants were randomly selected for fungal isolation, as all 20 diseased plants exhibited highly similar symptoms. Tissue segments from the interface between symptomatic and healthy root regions were excised. Surface sterilization followed these steps: roots were rinsed under running tap water to remove soil debris, immersed in 75% ethanol for 15 s, treated with 1% sodium hypochlorite (NaClO) for 3 min, rinsed three times in sterile distilled water, and dried on sterile filter paper. Sterilized root segments (approximately 3 mm^3^) were plated onto potato dextrose agar (PDA, Beijing Land Bridge Technology Co., Ltd., Beijing, China) medium. Tissues from each plant were distributed across three replicate plates (five pieces per plate), yielding 15 plates total. After incubation, the plant infection rate (Equation 2) and tissue colonization rate (Equation 3) were determined. After a 72-h incubation period at 25 °C, hyphal tips from emerging colonies were subcultured onto fresh PDA for purification. Pure isolates were cryopreserved in 15% glycerol at -80 °C and stored at 4 °C in the Strain Preservation Library, College of Pastoral Agriculture Science and Technology, Lanzhou University. One representative isolate was deposited at the China General Microbiological Culture Collection Center (CGMCC). Descriptions of taxonomic novelties were deposited in Fungal names (Wang et al., 2023).

(2) $$plant\;infection\;rate(\%)=\frac{number\;of\;plants\;yielding\;the\;target\;pathogen}{total\;number\;of\;examined\;plants}\times 100$$

(3) $$tissue\;colonization\;rate(\%)=\frac{number\;of\;\;pieces\;yielding\;the\;target\;pathogen}{total\;number\;of\;plated\;pieces}\times 100$$

### 2.2 Morphological characterization

Fungal cultures were established on PDA, Malt Extract Agar (MEA, Ararat Biotechnology Co., Ltd., China), and Oatmeal Agar (OMA; 20 g oatmeal, 20 g agar, 1000 mL H_2_O) media at 25 °C to characterize colony morphology and quantify radial growth rates. Hyphal structures were examined and measured under a light microscope (Leica DM4B, Leica Microsystems Wetzlar GmbH, Wetzlar, Germany).

### 2.3 DNA extraction, PCR amplification, sequencing, and phylogenetic analysis

Fungal genomic DNA was extracted from fresh mycelia using the E.Z.N.A.^®^ High Performance Fungal DNA Kit (Omega Bio-tek, Inc., GA, USA). Partial amplification of three target loci including ITS (internal transcribed spacer rDNA), LSU (large subunit), and *Tub2* (beta-tubulin gene) were performed using the following primer pairs: ITS1 (5’-TCCGTAGGTGAACCTGCGG-3’)/ITS4 (5’-TCCTCCGCTTATTGATATGC-3’) (White et al., 1990), LROR (5’-ACCCGCTGAACTTAAGC-3’)/LR5 (5’-ATCCTGAGGGAAACTTC-3’) (Vilgalys and Hester, 1990), and BTCadF (5’-MATGCGTGAAATYGTAAGT-3’)/BTCadR (5’-TCAGCACCCTCAGTGTAATG-3’) (Travadon et al., 2015). All primers were synthesized by Tsingke Biotechnology Co., Ltd (Beijing, China).

PCR reactions (25 μL) contained 12.5 μL 2×Taq PCR Master Mix (Biosharp Biotechnology Co., Ltd., Hefei, China), 9.5 μL double-distilled H_2_O, 1 μL each primer, and 1 μL template DNA. Amplification was performed in a BIO-RAD T100 thermal cycler (Bio-Rad Laboratories, Inc., CA, USA) with the following program: initial denaturation at 95 °C for 5 min; 35 cycles of denaturation at 95 °C for 30 s, annealing at 56 °C (ITS), 51 °C (LSU), or 53 °C (*Tub2*) for 30 s, and extension at 72 °C for 30 s; followed by a final extension at 72 °C for 10 min. Products were purified and bidirectionally sequenced by Tsingke Biotechnology Co., Ltd.

Sequences were assembled in SeqMan 5.0 (DNASTAR, Inc., WI, USA) and subjected to BLASTn searches against the National Center for Biotechnology Information (NCBI, https://www.ncbi.nlm.nih.gov/) database. Ex-type and ex-epitype reference sequences of target *Monosporascus* species were retrieved from GenBank (**Table 1**). Alignments were generated using MAFFT v. 7 online (https://mafft.cbrc.jp/alignment/server/) with the FFT-NS-i strategy (slow; interactive refinement method), trimmed in MEGA 7.0.2 (Tamura et al., 2013), and concatenated using Sequence Matrix 1.8 (Vaidya et al., 2011). Phylogenetic analyses employed Maximum Likelihood (ML) and Bayesian Inference (BI) on individual loci and the concatenated dataset. For ML, the best-fit model was selected using the Bayesian Information Criterion (BIC) via ModelFinder (Kalyaanamoorthy et al., 2017) in IQ-TREE v. 1.6.8 (Nguyen et al., 2015), with 1,000 bootstrap replicates. For BI, models were determined using MrModeltest 2.3 (Nylander, 2004) under the Akaike Information Criterion (AIC), and analyses were run in MrBayes 3.2.6 (Ronquist et al., 2012), with four chains for 1,000,000 generations (sampling every 1000 generations), discarding the first 25% as burn-in (convergence assessed by average standard deviation of split frequencies < 0.01). Trees were visualized in FigTree v. 1.4.4 (http://tree.bio.ed.ac.uk/software/figtree/) and edited in Adobe Acrobat Pro 2024 (Adobe Inc., CA, USA).

**Table 1:** Strains included in this study and their GenBank accession numbers.

Taxon	Strain	Host	Country	GenBank accessions			Reference
				ITS	LSU	*Tub2*	
*M. auratispora*	FMR 20333= CBS 149967	lagoon sediment	Spain	PP973385	PP973719	PP973383	(Barnés-Guirado et al., 2025)
*M. brasiliensis*	CMM-4837=MBr11	*Trianthema portulacastrum*	Brazil	MG735232	MG748801	MG725315	(Negreiros et al., 2019)
	CMM-4838=MBr12	*T. portulacastrum*	Brazil	MG735233	MG748802	MG725316	(Negreiros et al., 2019)
	CMM-4839=MBr13	*T. portulacastrum*	Brazil	MG735234	MG748803	MG725317	(Negreiros et al., 2019)
	CMM-4843=MBr17	*Boerhavia diffusa*	Brazil	MG735238	MG748807	MG725321	(Negreiros et al., 2019)
	CMM-4844=MBr18	*B. diffusa*	Brazil	MG735239	MG748808	MG725322	(Negreiros et al., 2019)
	CMM-4845=MBr20	*B. diffusa*	Brazil	MG735240	MG748809	MG725323	(Negreiros et al., 2019)
*M. bulgaricus*	P1811	*Microthlaspi perfoliatum*	Bulgaria	KT269083	–	–	(Crous et al., 2024a)
	P1916=CBS 151406	*Mi. perfoliatum*	Bulgaria	KT269184	PP454707	PP460994	(Crous et al., 2024a)
*M. caatinguensis*	CMM-4832=MBr6	*B. diffusa*	Brazil	MG735227	MG748796	MG725310	(Negreiros et al., 2019)
	CMM-4833=MBr7	*B. diffusa*	Brazil	MG735228	MG748797	MG725311	(Negreiros et al., 2019)
	CMM-4834=MBr8	*B. diffusa*	Brazil	MG735229	MG748798	MG725312	(Negreiros et al., 2019)
	CMM-4835=MBr9	*B. diffusa*	Brazil	MG735230	MG748799	MG725313	(Negreiros et al., 2019)
	CMM-4836=MBr10	*B. diffusa*	Brazil	MG735231	MG748800	MG725314	(Negreiros et al., 2019)
*M. cannonballus*	ATCC 26931	*-*	–	FJ430598	–	–	–
	CMM2386	*Cucumis melo*	Brazil	JQ771917	MG748825	JQ907303	(Negreiros et al., 2019)
	CMM2429	*Cu. melo*	Brazil	JQ762366	MG748826	JQ907311	(Negreiros et al., 2019)
	MC 0603	*Cu. melo*	Spain	JQ762364	MG748824	JQ907307	(Salem et al., 2013)
	MC 1103	*Cu. melo*	Spain	JQ762369	MG748823	JQ907302	(Salem et al., 2013)
*M. europaeus*	P1810=CBS 150022	*Mi. perfoliatum*	Bulgaria	KT269082	PP454705	PP481183	(Crous et al., 2024a)
	P1889	*Mi. perfoliatum*	Bulgaria	KT269158	PP454706	–	(Crous et al., 2024a)
*M. eutypoides*	CBS 132472	*-*	Tunisia	MH866020	MH877468	–	(Vu et al., 2019)
	CN020I9	*Stipagrostis* sp.	Namibia	ON074872	–	–	–
	CN021B1	*Stipagrostis* sp.	Namibia	ON074877	–	–	–
	MT 45	*Citrullus lanatus*	Tunisia	JQ958963	MG748827	JQ973834	(Salem et al., 2013)
	MT 47	*Ci. lanatus*	Tunisia	JQ958964	–	JQ973835	(Salem et al., 2013)
	NQ6GIII15	*Lycium barbarum*	China	MK183805	–	–	–
	R-21	*Cu. melo*	Iraq	OP554778	–	–	–
*M. ibericus*	CBS 110550	*-*	Spain	JQ 973832	MG748828	JQ973833	(Salem et al., 2013)
	ZMr17	*Stipa purpurea*	China	MK102683	–	–	(Lu et al., 2016)
***M. lespedezae* sp. nov.**	**LYZ1208 = CGMCC 3.29351**	***Lespedeza davurica***	**China**	**PX413139**	**PX413143**	**PX425268**	**This study**
	**LYZ1209**	***L. davurica***	**China**	**PX413140**	**PX413144**	**PX425269**	**This study**
	**LYZ1210**	***L. davurica***	**China**	**PX413141**	**PX413145**	**PX425270**	**This study**
	**LYZ1211**	***L. davurica***	**China**	**PX413142**	**PX413146**	**PX425271**	**This study**
*M. mossoroensis*	CMM-4856=MBr31	*T. portulacastrum*	Brazil	MG735251	MG748820	MG725334	(Negreiros et al., 2019)
	CMM-4857=MBr32	*T. portulacastrum*	Brazil	MG735252	MG748821	MG725335	(Negreiros et al., 2019)
	CMM-4858=MBr33	*T. portulacastrum*	Brazil	MG735253	MG748822	MG725336	(Negreiros et al., 2019)
*M. nordestinus*	CMM-4846=MBr21	*T. portulacastrum*	Brazil	MG735241	MG748810	MG725324	(Negreiros et al., 2019)
	CMM-4848=MBr23	*T. portulacastrum*	Brazil	MG735243	MG748812	MG725326	(Negreiros et al., 2019)
	CMM-4849=MBr24	*T. portulacastrum*	Brazil	MG735244	MG748813	MG725327	(Negreiros et al., 2019)
	CMM-4850=MBr25	*T. portulacastrum*	Brazil	MG735245	MG748814	MG725328	(Negreiros et al., 2019)
	CMM-4851=MBr26	*B. diffusa*	Brazil	MG735246	MG748815	MG725329	(Negreiros et al., 2019)
*M. semiaridus*	CMM-4827=MBr1	*T. portulacastrum*	Brazil	MG735219	MG748788	MG725302	(Negreiros et al., 2019)
	CMM-4828=MBr2	*T. portulacastrum*	Brazil	MG735220	MG748789	MG725303	(Negreiros et al., 2019)
	CMM-4829=MBr3	*T. portulacastrum*	Brazil	MG735221	MG748790	MG725304	(Negreiros et al., 2019)
	CMM-4830=MBr4	*T. portulacastrum*	Brazil	MG735222	MG748791	MG725305	(Negreiros et al., 2019)
	CMM-4860=MBr35	*B. diffusa*	Brazil	MG735225	MG748794	MG725308	(Negreiros et al., 2019)
*M. solitarius*	CBS150023	*Mi. perfoliatum*	Greece	KT269777	PP454708	PQ140140	(Crous et al., 2024b)
*Diatrype palmicola*	MFLU 15-0040	*Caryota urens*	Thailand	KP744438	KP744481	–	(Liu et al., 2015)
*Eutypa lata*	CBS 208.87	*Tilia* sp.	Switzerland	DQ006927	DQ836903	DQ006969	(Rolshausen et al., 2006)

- indicates missing information, novel species is indicated in bold.

### 2.4 Fulfillment of Koch’s postulates

Pathogenicity was confirmed using a representative isolate on *L. davurica* seedlings. Seeds were scarified with fine sandpaper to break dormancy, surface-sterilized (30 s in 75% ethanol, 1 min in 2% NaClO), and rinsed three times in sterile distilled water. Germination occurred on sterile moist filter paper in Petri dishes. Seedlings with roots approximately 1 cm in length were inoculated by pressing the root tip onto the margin of a 2-cm-diameter actively growing PDA colony of the isolate. Controls used sterile pure PDA. After 2 days, 10 seedlings were transplanted per pot (four pots per treatment) into autoclaved loam soil (dry-heat sterilized at 165 °C for 2 h). The experiment was repeated independently. Plants were grown in a greenhouse at 25 ± 2 °C and 80% relative humidity. Disease progression was monitored, and final assessments included disease incidence (Equation 1), disease index (Equation 4), and growth parameters (plant height from soil surface to apex; root length from base to tip). Disease severity was rated according to the scale in **Table 2**.

**Table 2:** The standard of severity levels.

Severity levels	Symptom
0	No discoloration or rot in the roots.
1	Discolored or rotted root area accounted for 0% to 10% of the total root system
2	Discolored or rotted root area accounted for 11% to 30% of the total root system
3	Discolored or rotted root area accounted for 31% to 50% of the total root system.
4	Discolored or rotted root area accounted for more than 50% of the total root system.

(4) $$disease\;index=\frac{\sum (number\;of\;plants\;in\;each\;severity\;scale\;\times \;corresponding\;scale\;value)}{highest\;scale\;value\;\times \;total\;number\;of\;plants}\times 100$$

### 2.5 Fungicide sensitivity assay

Sensitivity of a representative isolate was tested against six fungicides previously effective (>60%) against *M. cannonballus* in melon sudden wilt studies (Cohen et al., 1999). Tested products (Guoguang Agrochemical Co., Ltd., Sichuan, China) were carbendazim (50% active ingredient), diniconazole (12.5% a.i.), hexaconazole + ethirimol (30% total a.i.), mancozeb (80% a.i.), quintozene (40% a.i.), and thiophanate-methyl (50% a.i.). A preliminary assay at 10 μg/mL a.i. determined baseline inhibition and informed concentration gradients: carbendazim (0.2, 1, 2, 3, and 4 μg/mL a.i.), diniconazole (0.2, 1, 2, 5, and 10 μg/mL a.i.), hexaconazole + ethirimol (1, 2, 5, 10, and 20 μg/mL a.i.), mancozeb (10, 20, 40, 80, and 160 μg/mL a.i.), quintozene (10, 20, 30, 40, and 80 μg/mL a.i.), and thiophanate-methyl (2, 4, 10, 20, and 40 μg/mL a.i.). Fungicide-amended PDA was prepared, while controls lacked fungicide. Mycelial plugs (5-mm-diameter) from colony margins were placed centrally on plates (five replicates per concentration; two independent runs). After 7 days at 25 °C, colony diameters were measured in two perpendicular directions. Mycelial growth inhibition rate (Equation 5) was calculated (Ji et al., 2019). EC_50_ values were estimated using a four-parameter log-logistic model in GraphPad Prism v. 8.0.2 (GraphPad Software, CA, USA).

(5) $$mycelial\;growth\;inhibition\;rate(\%)=(1-\frac{mean\;diameter\;of\;treated\;colonies}{mean\;diameter\;of\;control\;colonies})\times 100$$

### 2.6 Statistical analysis

Analysis Photographs were processed in Adobe Photoshop CC 2015 (Adobe Inc., CA, USA). Fungicide data were managed in Excel 2021 and visualize in GraphPad Prism 8.0.2. Growth parameters were analyzed in SPSS 21.0 (IBM, NY, USA) using independent t-tests (means ± SEM, significance at *P* < 0.001).

## 3 Results

### 3.1 Disease symptoms

**Figure 1:**
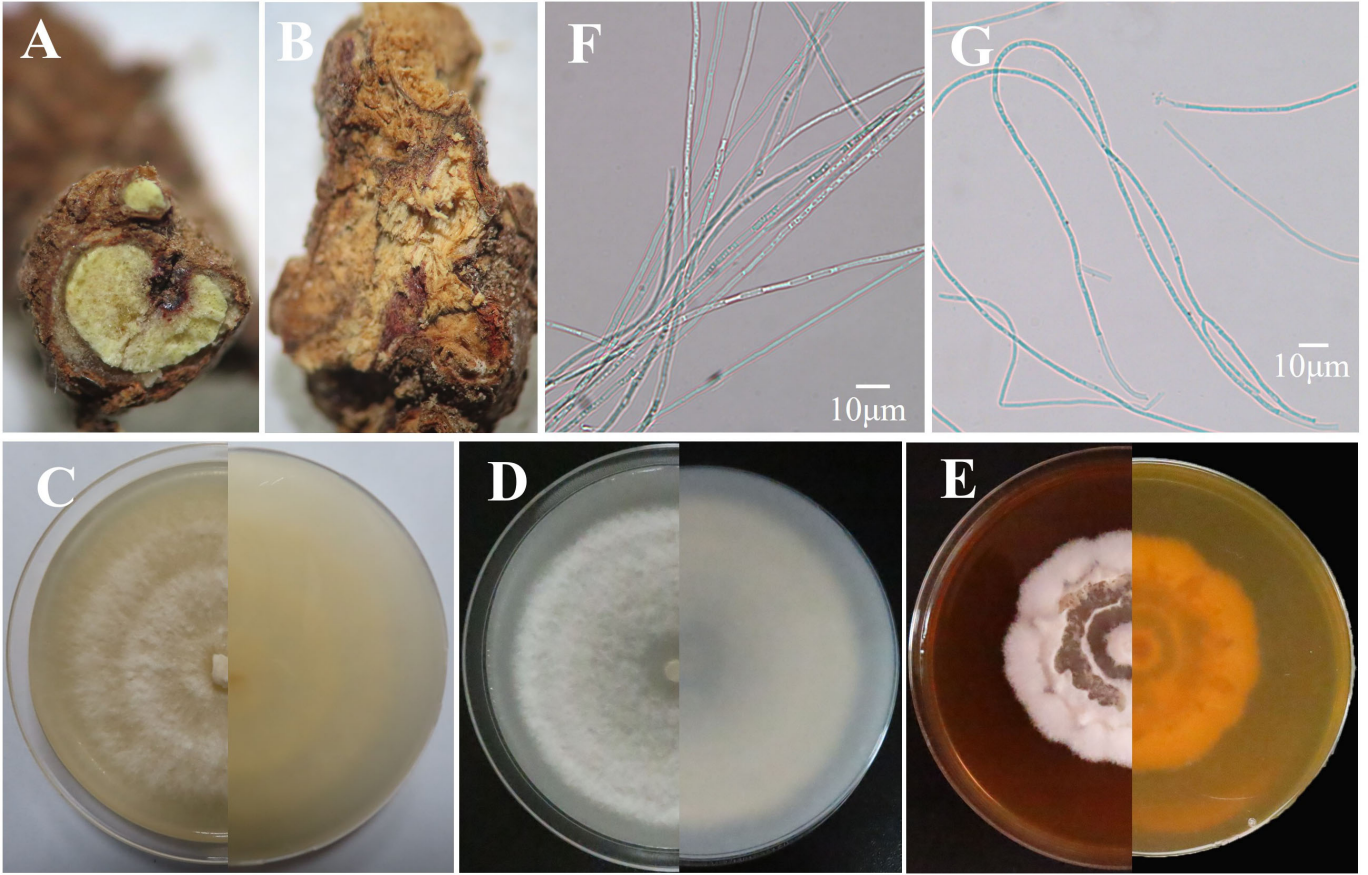
Field symptoms of root rot on *Lespedeza davurica* caused by *Monosporascus lespedezae* sp. nov. and morphological characteristic of LYZ1208 (CGMCC 3.29351). **(A, B)** Field symptoms. **(C)** Upper and reverse colony morphology on PDA after 9-day incubation at 25°C. **(D)** Upper and reverse colony morphology on OA after 9-day incubation at 25°C. **(E)** Upper and reverse colony morphology on MEA after 9-day incubation at 25°C. **(F, G)** Hyphal structure exhibiting branching and septation, scale bar = 10 μm.

Symptomatic roots of *L. davurica* showed brown discoloration upon excavation and transverse sectioning. Initial symptoms appeared as faint brown lesions restricted to the root cortex adjacent to the epidermis, whereas healthy tissues remained white to pale yellow (**Figure 1A**). Advanced symptoms showed light brown to black discoloration throughout the entire cortex and stele (**Figure 1B**). All 20 plants collected from the surveyed site displayed these symptoms, resulting in a disease incidence of 100%. Fungi with uniform morphology were consistently isolated from symptomatic root tissues, achieving a plant infection rate of 100% (5/5 plants) and a tissue colonization rate of 100% (75/75 tissue pieces). Four representative isolates (LYZ1208, LYZ1209, LYZ1210, and LYZ1211) were purified and preserved. Isolate LYZ1208 (deposited as CGMCC 3.29351) was designated the representative strain for subsequent experiments.

### 3.2 Morphological characteristic

On PDA, colonies exhibited a mycelial growth rate of 5.16 ± 0.06 mm/d at 25 °C. They were slightly sparse, round, with well-defined concentric rings and minimal hyphae at the junctions, sporulation absent; from above side, the colony appeared milky white, the reverse side was white, and no soluble pigment was produced (**Figure 1C**). Colonies on OA with a mycelial growth rate of 4.26 ± 0.05 mm/d at 25 °C, featured a cottony texture with luxuriant hyphae, round, concentric ring, and well-defined margins, sporulation absent; from above side, the primary zone was white, with hyphae gradually disappearing in aging zones, the reverse side exhibited the same coloration, and soluble pigment absent (**Figure 1D**). On MEA, colonies grew more slowly than on PDA or OA, with a mycelial growth rate of 3.74 ± 0.09 mm/d at 25 °C. The mycelium formed cotton-like tufts distributed at the colony margin, which was irregular and wavy but still well-defined, sporulation absent, and the colony appeared white both from the above and reverse side, with no soluble pigment produced (**Figure 1E**). Hyphae were branched, septate, smooth, hyaline, thin-walled, 1.5-3 µm wide (**Figures 1F, G**).

### 3.3 Phylogenetic analysis

Gene sequences of the ITS, LSU, and *Tub2* loci from isolates LYZ1208 - LYZ1211 were deposited in GenBank (ITS: PX413139 - PX413142; LSU: PX413143 - PX413146; *Tub2*: PX425268 - PX425271). BLASTn searches indicated high similarity to *Monosporascus* spp. *Diatrype palmicola* (MFLU 150040) and *Eutypa lata* (CBS 208.87) were used as outgroups. The concatenated alignment (ITS + LSU + *Tub2*) included 1,924 positions (ITS: 528 bp; LSU: 683 bp; *Tub2*: 713 bp).

Phylogenetic analyses revealed that isolates LYZ1208 (CGMCC 3.29351), LYZ1209, LYZ1210, and LYZ1211 formed a monophyletic clade with representative *Monosporascus* strains in both single-locus and concatenated multigene trees. For phylogenetic reconstruction, the best-fit model selected by ModelFinder for the ML tree of the concatenated dataset was TIM2ef+I+G. In contrast, the models selected by MrModeltest for the BI trees were SYM+I+G for ITS, GTR+I+G for LSU, and TIM1+I+G for *Tub2*. Phylogenetic nodes displayed both Bayesian posterior probabilities (PP ≥ 0.9) and ML bootstrap support values (ML ≥ 70%).

**Figure 2:**
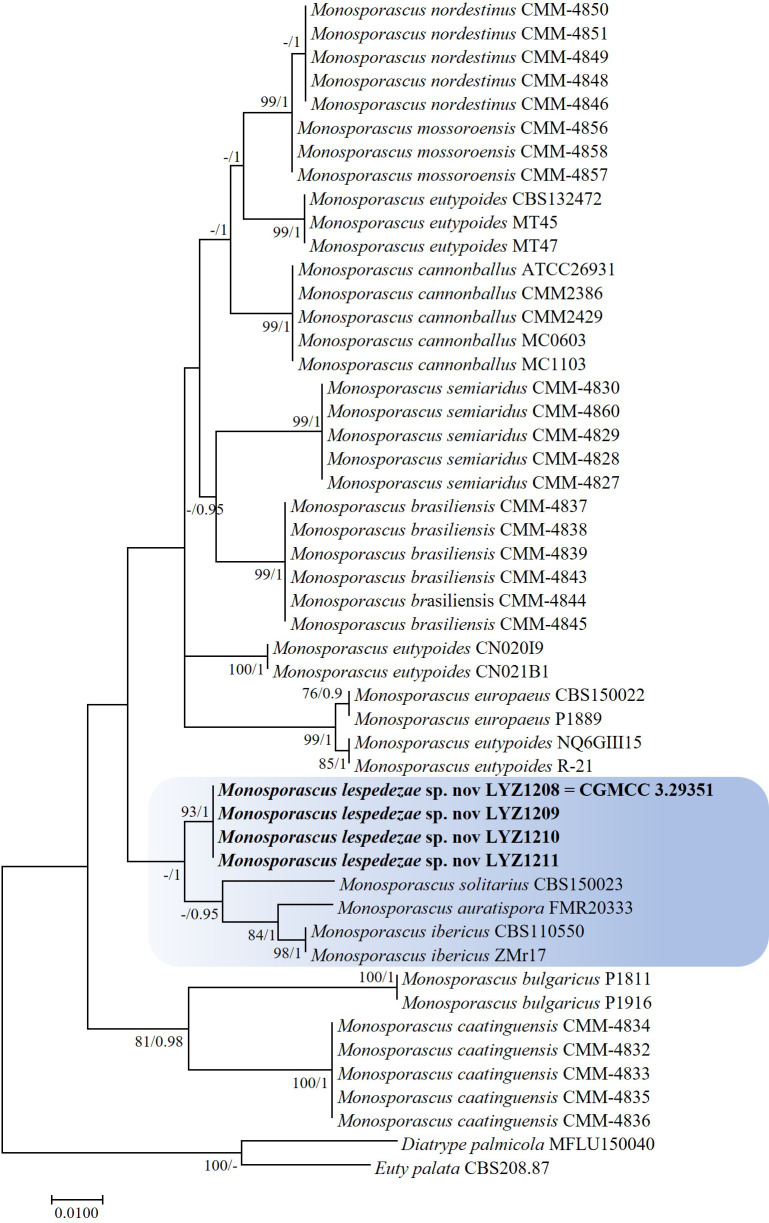
Maximum likelihood phylogenetic tree obtained from the concatenated *ITS*, *LSU* and *Tub2* sequences of *Monosporascus* (*Diatrypaceae*, *Sordariomycetes*) showing the position of *Monosporascus lespedezae* sp. nov. within the genus. Maximum Likelihood bootstrap support (BS) values and Bayesian posterior probabilities (PP) are shown at the nodes. Branches with 100% BS/1 PP are indicated as “*”. Branches with less than 70% BS/0.9 PP are indicated as “-”. Novel species is indicated in bold; type species in blue.

**Figure 3:**
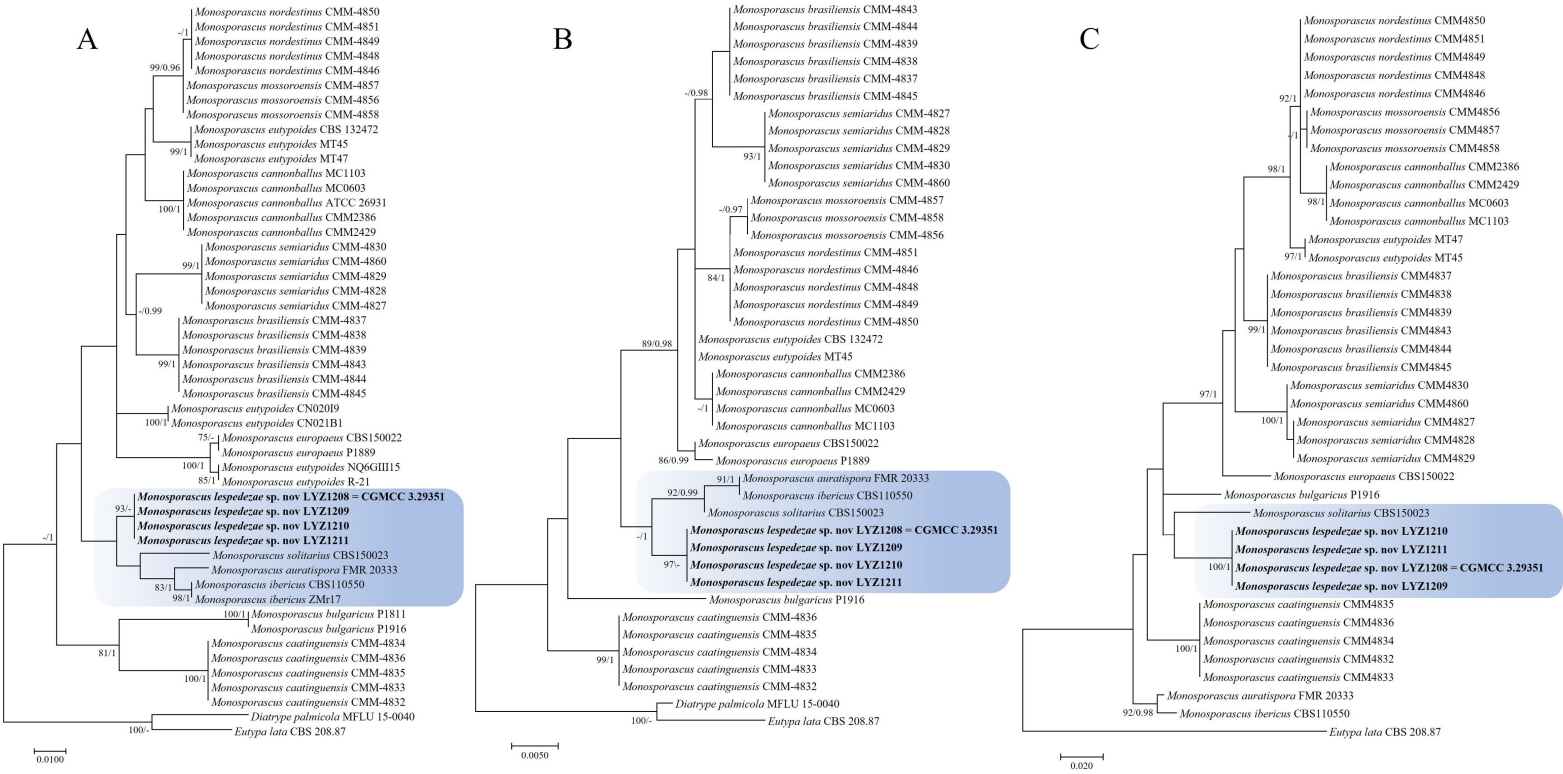
Maximum likelihood analysis tree of the single-locus analyses including *ITS*, *LSU*, and *Tub2* of *Monosporascus* (*Diatrypaceae, Sordariomycetes*) showing the position of *Monosporascus lespedezae* sp. nov. within the genus. **(A)** Maximum likelihood analysis tree of ITS sequences. **(B)** Maximum likelihood analysis tree of LSU sequences. **(C)** Maximum likelihood analysis tree of *Tub2* sequences. Maximum likelihood bootstrap support (BS) values and Bayesian posterior probabilities (PP) are shown at the nodes. Branches with 100% BS/1 PP are indicated as “*”. Branches with less than 70% BS/0.9 PP are indicated as “-”. Novel species is indicated in bold; type species in blue. The single loci ITS, LSU, and *Tub2* separated *M. lespedezae* sp. nov. from the other 12 species in *Monosporascus*.

In the concatenated phylogenetic tree, *M. lespedezae* sp. nov. resolved as distinct lineage within *Monosporascus* with robust support (ML = 86%, PP = 1) and sister to a clade comprising *M. auratispora*, *M. ibericus*, and *M. solitarius* (**Figure 2**). Single-locus phylogenies (ITS, LSU, *Tub2*) independently confirmed the separation of *M. lespedezae* sp. nov. from the remaining 12 recognized *Monosporascus* species (**Figure 3**).

### 3.4 Pathogenicity tests

**Figure 4:**
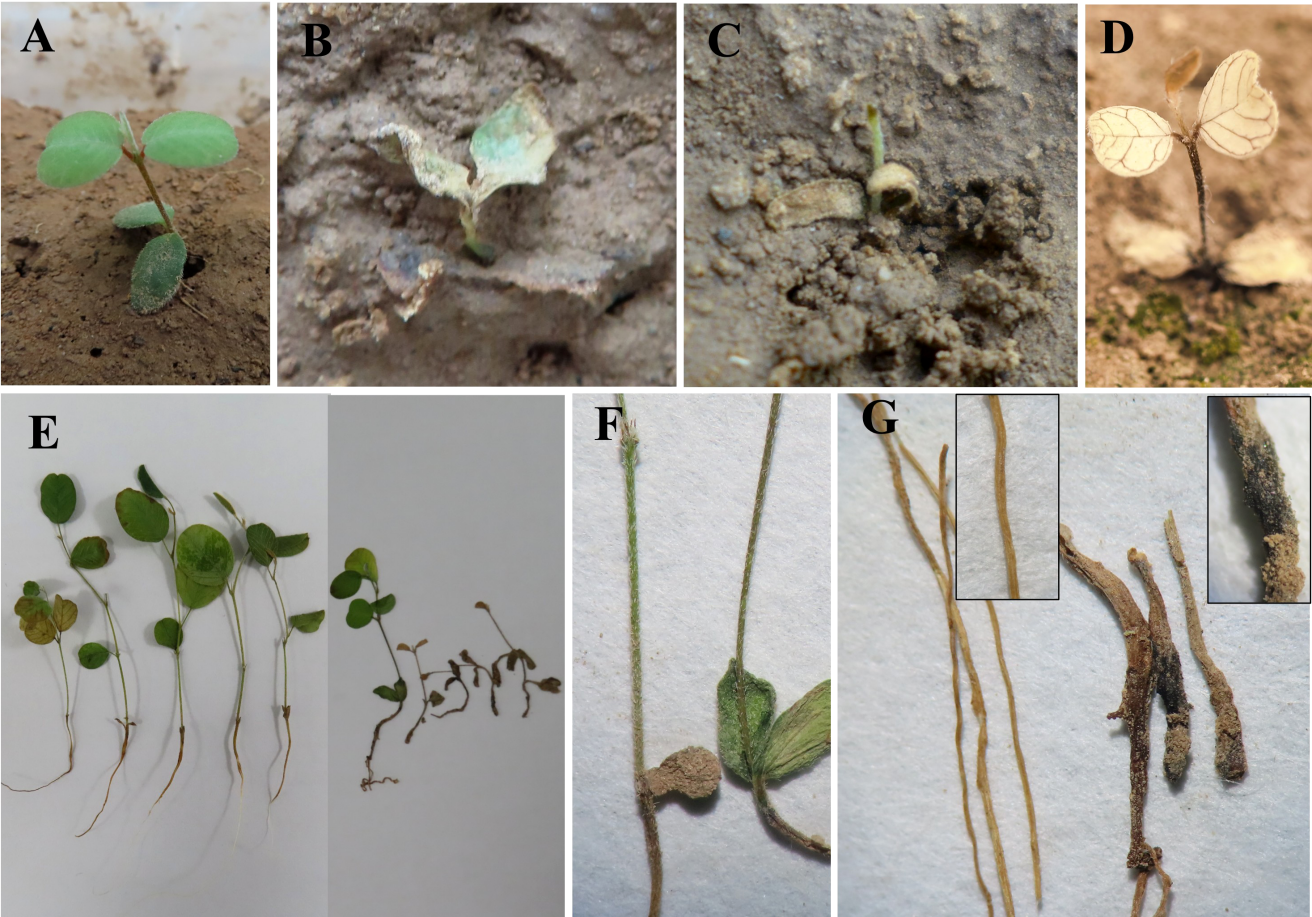
Inoculated symptoms of root rot on *Lespedeza davurica* caused by *Monosporascus lespedezae* sp. nov. **(A)** Foliar symptoms of control. **(B–D)** Disease progression. **(B)** Cotyledon wilting; **(C)** Aborted true leaf development; **(D)** Systemic desiccation of entire plant. **(E)** Entire symptoms of control (left) and inoculated (right) plants. **(F)** Crown symptoms of control (left) and inoculated (right) plants. **(G)** Root symptoms of control (left) and inoculated (right) plants.

Non-inoculated control plants remained asymptomatic, producing green true leaves throughout the experiment (**Figure 4A**). In contrast, inoculated plants exhibited abnormal symptoms at 9 days post-transplantation (dpt): cotyledon edges yellowed, withered, and curled (**Figure 4B**); some exhibited complete cotyledon death that blocked true leaf emergence despite continued stem elongation and yellowing (**Figure 4C**). Approximately 10% appeared normal until the second true leaf stage, then yellowed, withered, and died progressively from the base upward (**Figure 4D**). Excavated inoculated plants displayed markedly reduced growth relative to controls (**Figure 4E**). Control stem bases remained green, whereas surviving inoculated stems turned light yellowish-brown (**Figure 4F**). Control roots were healthy, light-yellow, and slender, while inoculated roots showed brown-to-black discoloration and swelling (**Figure 4G**). The fungus was successfully re-isolated from symptomatic roots of inoculated plants and confirmed as the original isolate through morphological and molecular analysis, thereby fulfilling Koch’s postulates.

**Figure 5:**
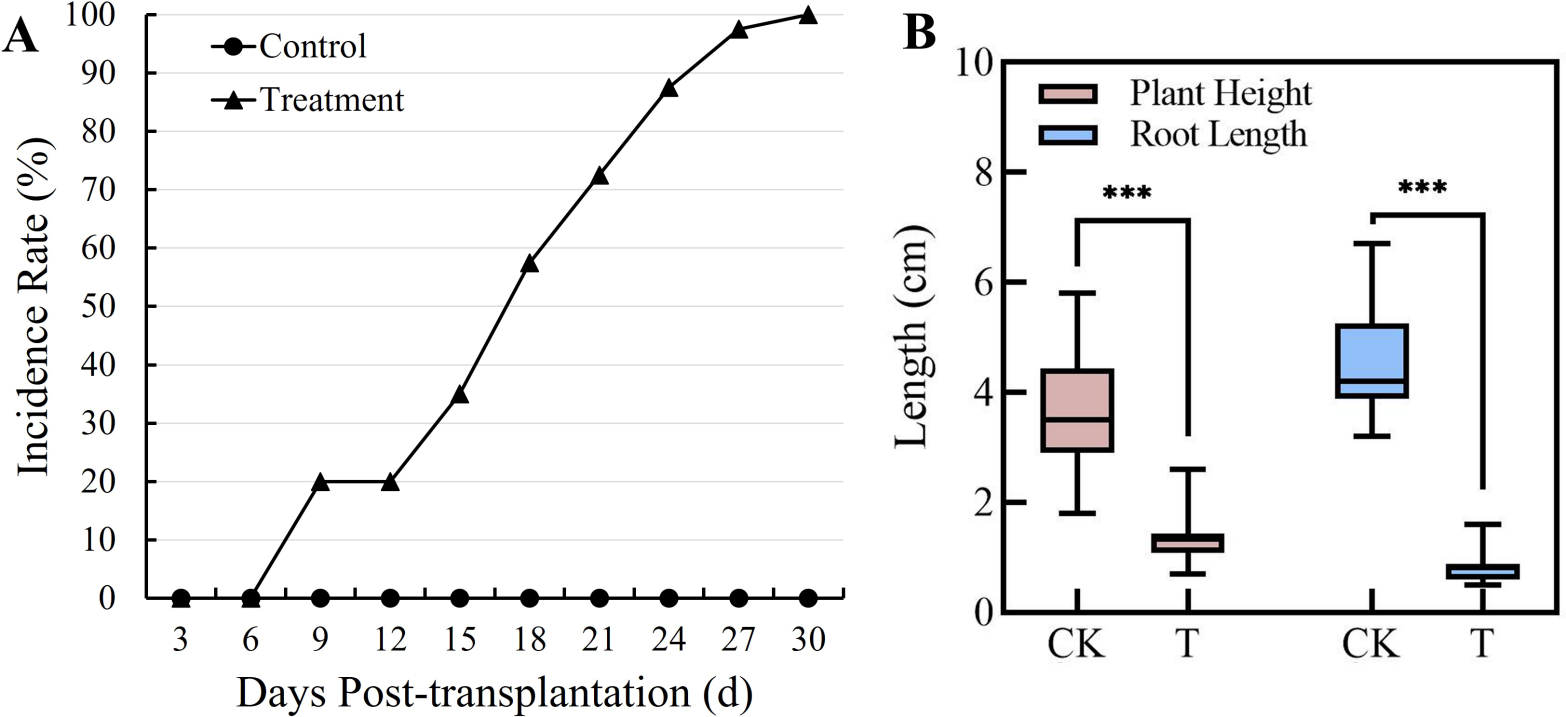
Effects of inoculated *Monosporascus lespedezae* sp. nov. on the incidence rate and growth traits of *Lespedeza davurica*. **(A)** The changing trends of the incidence rate over time in the control and treatment group after transplanting. **(B)** Box-plot comparison of plant height (in red) and root length (in blue) between the control and treatment group. *** indicates an extremely significant difference between the control and the treatment group (*P* < 0.001).

By 30 dpt, the disease incidence reached 100% (**Figure 5A**), with a disease index of 98.13. Measurement results showed that control plant height (3.70 ± 1.13 cm) was extremely significantly higher compared to inoculated plants (1.37 ± 0.49 cm) (*P* < 0.001), and control root length (4.50 ± 1.00 cm) was extremely significantly higher relative to inoculated roots (0.84 ± 0.30 cm) (*P* < 0.001) (**Figure 5B**).

### 3.5 Fungicide sensitivity test

At 10 μg/mL a.i., inhibition rates against *M. lespedezae* sp. nov. diverged from those reported for *M. cannonballus* (**Table 3**). Carbendazim and diniconazole showed increased inhibition rates (from 95.7% to 100% and from 77.5% to 95.85%, respectively), while hexaconazole + ethirimol and thiophanate-methyl were comparable (76.84% vs. 77.5%; 64.61% vs. 66.5%). Mancozeb and quintozene showed substantially lower inhibition (17.56% vs. 83.2%; 5.39% vs. 66.5%).

**Table 3:** Inhibition rates of six fungicides at 10 μg/mL a.i.

Fungicide	Inhibition rate (%)
carbendazim	100
diniconazole	95.85
hexaconazole + ethirimol	76.84
thiophanate-methyl	64.61
mancozeb	17.56
quintozene	5.39

**Figure 6:**
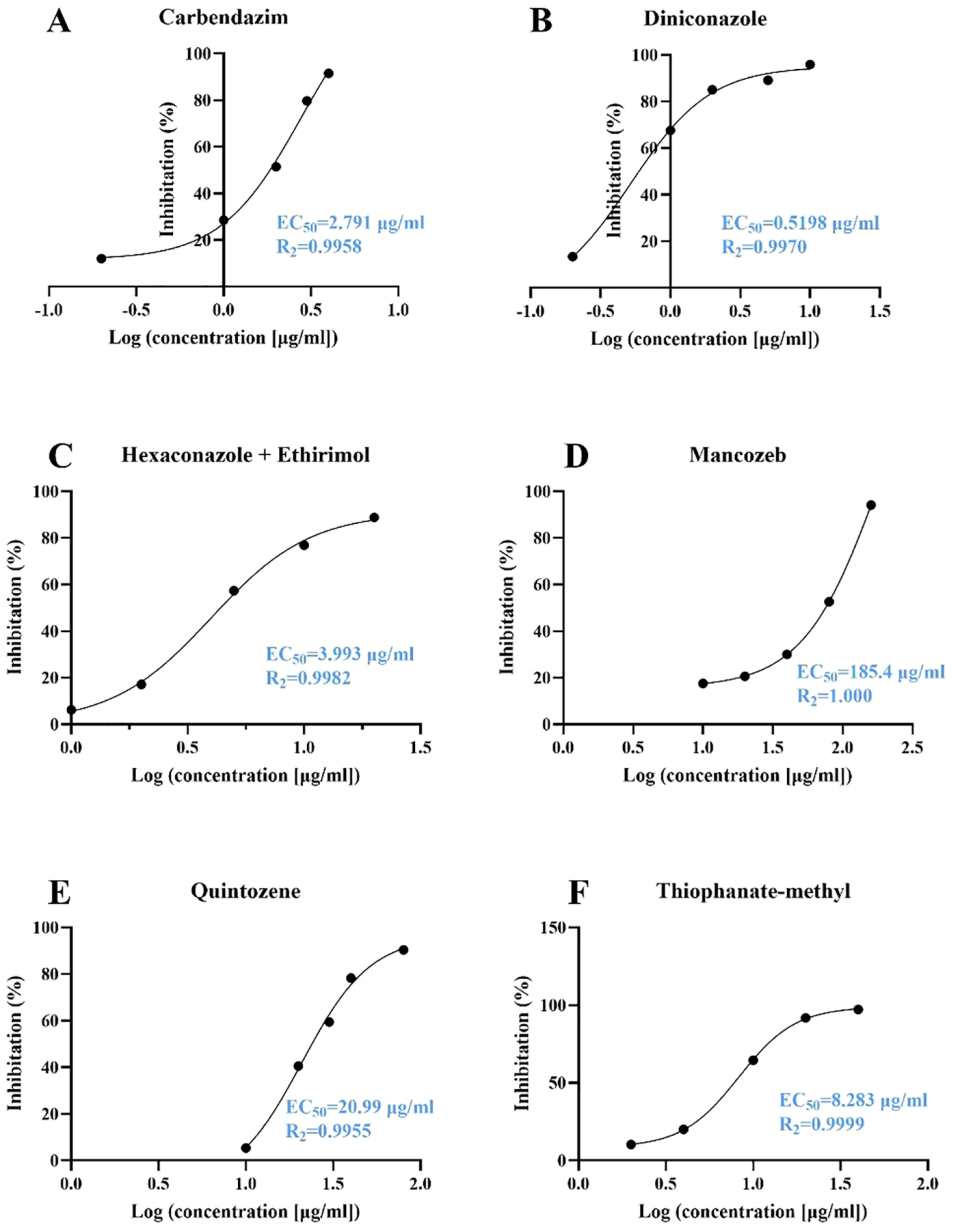
Four parameter log-logistic estimated EC_50_ values for the *Monosporascus lespedezae* sp. nov. isolate causing root rot on *Lespedeza davurica*, against chemical fungicides used to manage root rot on *Lespedeza davurica* in China: **(A)**, Carbendazim; **(B)** Diniconazole; **(C)**, Hexaconazole + Ethirimol; **(D)**, Mancozeb; and **(E)**, Quintozene; **(F)**, Thiophanate-methyl.

*In vitro* mycelial growth inhibition assays revealed that all six fungicides suppressed the growth of *M. lespedezae* sp. nov., albeit with differing potency (**Figure 6**). Diniconazole exhibited the strongest suppression (EC_50_ = 0.5198 μg/ml a.i.). Carbendazim, hexaconazole + ethirimol, thiophanate-methyl, and quintozene showed moderate effects (EC_50_ 2.791 - 20.99 μg/ml a.i.). Mancozeb was least inhibitory (EC_50_ = 185.4 μg/ml a.i.).

## 4 Taxonomy

*Monosporascus lespedezae* T.T. Wang, & Y.Z. Li, sp. nov.

Fungal Names accession number: FN 573602

*Etymology*. *Monosporascus lespedezae* (Latin) refers to the representative isolate that was originally identified from the host genus (*Lespedeza davurica*).

*Classification*: *Diatrypaceae*, *Xylariales*, *Sordariomycetes*.

*Habitat and distribution*: Root pathogen, currently known only from China.

*Typus*: CHINA, Gansu Province, Huan County, 106°49’52.09” E, 37°7’22.98” N, 1538.4 m, isolated from symptomatic root of *Lespedeza davurica*, 18/08/2023, collected and isolated by Tong-Tong Wang and Yan-Zhong Li. Holotype: LYZ1208 (permanently preserved in a metabolically inactive state at CGMCC 3.29351; culture ex-type CGMCC 3.29351; ITS, LSU, and *Tub2* sequences GenBank PX413139, PX413143, and PX425268).

*Additional material examined*: CHINA, Gansu Province, Huan County, 106°49’52.09” E, 37°7’22.98” N, 1538.4 m, isolated from symptomatic root of *Lespedeza davurica*, 18/08/2023, collected and isolated by Tong-Tong Wang and Yan-Zhong Li, (LYZ1209, ITS, LSU, and *Tub2* sequences GenBank PX413140, PX413144, and PX42569; LYZ1210, ITS, LSU, and *Tub2* sequences GenBank PX413141, PX413145, and PX425270; LYZ1211, ITS, LSU, and *Tub2* sequences GenBank PX413142, PX413146, and PX425271).

*Cultural characteristics*. As described in the morphological characteristics section above.

*Notes*. *M. lespedezae* sp. nov. can be distinguished from its phylogenetically closest species, *M. auratispora*, *M. ibericus*, and *M. solitarius*, based on cultural characteristics. On PDA and OA media, all four species form white, fast-growing asexual colonies, but *M. lespedezae* sp. nov. uniquely exhibits distinct concentric zonation. On MEA medium, the phylogenetic relatives present as flat colonies with diffused margins, whereas *M. lespedezae* sp. nov. develops fluffy cotton-like mycelial tufts with well-defined margins. Mycelium morphology fails to differentiate these taxa, as all share branched, septate, smooth, and hyaline hyphae. Notably, *M. lespedezae* sp. nov. (1.5 - 3 μm) displays significantly wider hyphae compared to *M. auratispora* (1.0 μm), while being comparable in width to *M. ibericus* (2 - 4 μm) and *M. solitarius* (1.5 - 4 μm). Unfortunately, the sexual state of *M. lespedezae* sp. nov. remains unobserved in this study.

Based on a megablast search of NCBI database, the closest hits using the ITS sequence had highest similarity to *Monosporascus* sp. [isolate SDQ-25, GenBank OQ398850.1, Identities = 499/505 (99%), three gaps (0%)], *Monosporascu*s sp. [isolate HO7, GenBank OQ430658.1, Identities = 495/506 (98%), two gaps (0%)], and *Fusarium neerlandicum* [isolate 17fn, GenBank PV400024.1, Identities = 487/497 (98%), six gaps (1%)]. Closest hits using the LSU sequence are *Monosporascus* sp. [isolate P2549, GenBank PP454708.1, Identities = 673/681 (99%), no gaps], *Monosporascus auratispora* [strain FMR 20333, GenBank PP973719.1, Identities = 672/682 (99%), two gaps (0%)], and Xylariales [isolate P2581, GenBank KP114327.1, Identities = 670/682 (98%), two gaps (0%)]. Closest hits using the *Tub2* sequence are *Monosporascus caatinguensis* [isolate MBr6- MBr10, GenBank MG725310.1 - MG725314.1, Identities = 628/679 (92%), seven gaps (1%)], *Monosporascus ibericus* [strain CBS 110550, GenBank JQ973833.1, Identities = 630/682 (92%), twelve gaps (1%)], and *Monosporascus brasiliensis* [isolate MBr11 - MBr18, GenBank MG725315.1- MG725322.1. Identities = 617/680(91%), one gap (0%).

## 5 Discussion

In 2012, researchers concluded that all *Monosporascus* species are soilborne and generally adapted to hot, arid, or semiarid climates with saline and alkaline soils (Cohen et al., 2012). This pattern holds for several species: *M. brasiliensis*, *M. caatinguensis*, *M. mossoroensis*, *M. nordestinus*, and *M. semiaridus* were isolated from *Trianthema portulacastrum* and *Boerhavia diffusa* in northeastern Brazil (MAT 28°C), a hot semiarid region where both hosts are salt- and alkali-tolerant (Negreiros et al., 2019). Similarly, *M. auratispora* and *M. ibericus* have been reported from saline environments in Spain (Barnés-Guirado et al., 2025; Collado et al., 2002). However, recent studies have begun to challenge this ecological constraint. Species such as *M. bulgaricus* and *M. europaeus* were isolated from *Microthlaspi perfoliatum* roots in Bulgaria, a temperate continental climate zone (MAT 17°C) with low drought risk and non-saline soils (Crous et al., 2024a). Similarly, *M. solitarius* was obtained from the same host in Greece, a Mediterranean climate region (MAT 22°C) (Crous et al., 2024b). In this study, *M. lespedezae* sp. nov. was isolated from *L. davurica* (Fabaceae) roots in a semiarid region with alkaline soils and low temperatures (MAT 7.5 °C) (Cao et al., 2020). Collectively, these findings indicate that *Monosporascus* exhibits a broad ecological adaptability, ranging from classical hot−arid saline−alkaline environments, temperate and Mediterranean climates, as well as low−temperature semiarid zones. Moreover, the host range now includes Fabaceae, expanding beyond previously reported Brassicaceae and Amaranthaceae hosts. Together, these observations highlight the contribution of host association and soil microhabitat, in addition to climate, in shaping the distribution of *Monosporascus* species. All isolates recovered from symptomatic roots were consistently identified as *M. lespedezae* sp. nov., supporting its primary role in the observed disease outbreak. Nevertheless, because sampling was limited to a small number of plants from a single site, the geographic distribution and population structure of this pathogen remain unresolved. Broader surveys across regions, host plants, and environmental conditions will be required to determine whether it is endemic to the Loess Plateau or more widely distributed, and to evaluate its potential for spread.

The newly identified species, *M. lespedezae* sp. nov., did not produce reproductive structures under the tested conditions, a characteristic frequently observed within the genus. Notably, approximately half of the species in this genus have only been observed as colonies or mycelial structures, without reproductive structures. Consequently, their taxonomy relies entirely on molecular phylogenetic analysis. For example, *M. bulgaricus*, *M. europaeus*, and *M. solitarius* are morphologically indistinguishable by traditional methods, but were delimited as distinct species through integrative multilocus analysis of ITS, LSU, and *Tub2* sequences (Crous et al., 2024a, b). This study employed a similar multi-gene integrative strategy to establishing the status of *M. lespedezae* sp. nov. in the absence of definitive morphological characteristics. Quantitatively, *M. lespedezae* sp. nov. exhibits 1-2% sequence divergence in ITS and LSU regions and 8-9% in *Tub2* compared to its closest relatives, supporting its recognition as a distinct species. This pattern underscores substantial cryptic diversity within the genus. Temporal analysis further supports this: 9 of the 14 currently recognized taxonomic units (64%) have been described since 2019. This surge in taxonomic updates strongly supports the hypothesis of substantial cryptic diversity awaiting discovery (Negreiros et al., 2019; Robinson et al., 2020). This reflects a general trend in fungal systematics, where advances in phylogenetics and genomics are enabling the resolution of many lineages that were previously difficult to delimit or remained undiscovered by traditional culturing and morphological methods (Hibbett et al., 2007; Hyde et al., 2024). However, the absence of a sexual stage in *M. lespedezae* sp. nov. highlights a common but important gap in *Monosporascus* biology, where reproductive modes remain unclear for many taxa. Further investigation into environmental or host-related triggers for sexual reproduction will be essential to clarify its life history and epidemiological behavior.

Soilborne pathogens adversely impact plant diversity, alter soil microbial diversity, and impair disease suppressiveness, ultimately degrading soil health and ecosystem sustainability (Gómez-Aparicio et al., 2022; Singh et al., 2025). The extensive habitats and abundant companion plants of *L. davurica* pose dual challenges for *M. lespedezae* sp. nov. First, it may intensify soilborne risks, as established soil pathogen suppressiveness can persist for decades (Schlatter et al., 2017), and the genus’s salt-alkali tolerance could allow accumulation in alkaline conditions over time. Second, it expands host range, with companion plants acting as temporary inoculum reservoirs (Dong and Zhou, 2022). Because this genus infects Brassicaceae, Cucurbitaceae, and Poaceae (Crous et al., 2024a; Lu et al., 2016; Negreiros et al., 2019), companion plants could act as alternative hosts or reservoirs, thereby facilitating cross-species transmission. In addition, the temporal dynamics of how *M. lespedezae* sp. nov. influences rhizosphere microbial community composition and function remain unclear. Long-term studies integrating microbiome analyses will be essential to elucidate how this pathogen shapes microbial interactions, community succession, and disease suppressiveness over time.

Fungicide sensitivity testing provides the initial step toward effective disease management. This study provides the first comparative analysis of *in vitro* sensitivity between the novel *M. lespedezae* sp. nov. and the well-studied *M. cannonballus* (Cohen et al., 1999). Both benzimidazole (carbendazim) and demethylation inhibitor (diniconazole) fungicides showed consistently high activity against both species. In contrast, mancozeb and quintozene exhibited markedly lower inhibition against *M. lespedezae* sp. nov. (17.56% and 5.39%, respectively, vs. 83.2% and 66.5% for *M. cannonballus*). High EC_50_ values for mancozeb and quintozene should be interpreted cautiously, as standard mycelial growth assays have inherent limitations for evaluating protectant fungicides, which primarily form surface barriers rather than inhibit established growth (Gullino et al., 2010; Russell, 2005). Their field efficacy depends on application timing, coverage, rates, and persistence rather than *in vitro* mycelial inhibition (Cohen et al., 1999; Gullino et al., 2010). The low EC_50_ values for carbendazim and diniconazole indicate strong target-site specificity and potent disruption of fungal processes, positioning them as priority candidates for further evaluation. Cross-species consistency in carbendazim sensitivity (>95% inhibition at 10 μg/mL) suggests conserved efficacy of methyl benzimidazole carbamates against *Monosporascus* spp., attributable to their action on β-tubulin (Davidse, 1973; Russell, 1995; Xu et al., 2019). In many ascomycetes, point mutations in the β-tubulin gene confer resistance to methyl benzimidazole carbamate (MBC) fungicides. Specifically, substitutions at codon 198 (Glu to Ala/Lys) are associated with high-level resistance, while substitution at codon 200 (Phe to Tyr) confers moderate resistance (Amini et al., 2023; Cortaga et al., 2023; Yin et al., 2023). Although the β-tubulin gene of *Monosporascus* species has not yet been characterized, the conservation of these resistance mutations across diverse fungi suggests that sustained carbendazim application may lead to resistance development in *M. lespedezae* sp. nov. Therefore, future resistance management should prioritize monitoring mutations at codons 198 and 200. The notably higher sensitivity of *M. lespedezae* sp. nov. to diniconazole compared with *M. cannonballus* may reflect interspecific differences in sterol biosynthesis, uptake or metabolism (Cools and Fraaije, 2013; Ma and Michailides, 2005), warranting studies on potential DMI resistance. These results establish a scientific baseline rather than direct field recommendations. Carbendazim and diniconazole emerge as promising for subsequent *in vivo* pot trials and field tests using commercial formulations under local conditions. To build a comprehensive sensitivity baseline for resistance monitoring, future work should examine natural variation across a broader geographic collection of isolates (Ma and Michailides, 2005).

In conclusion, this study identifies *M. lespedezae* sp. nov. as a novel soilborne pathogen associated with root rot of *L. davurica* and expands the ecological and host range of *Monosporascus*. While this work provides a foundation through taxonomic and pathogenicity characterization, key aspects including life cycle, distribution, host range, and virulence mechanisms (such as the potential roles of cell wall-degrading enzymes, phytotoxins, secreted effectors, and host immune evasion strategies) remain to be elucidated. In particular, comparative genomic analyses between pathogenic and endophytic *Monosporascus* species can help elucidate the genetic determinants underlying virulence and host adaptation, thereby informing targeted disease management strategies and resistance breeding. Notably, host-pathogen interactions may involve complex signaling processes. Reactive oxygen species function both as defense signals and contributors to cellular damage, potentially explaining symptom progression from cortical browning to vascular discoloration (Ali et al., 2025). The integration of single-cell and spatial transcriptomics will enable resolution of cell-type-specific responses and provide deeper mechanistic insight into disease development (Ali et al., 2026). Addressing these gaps will be essential for understanding the ecological impact of this pathogen and for developing effective disease management strategies to mitigate its impact on grassland ecosystems.

## Data Availability

The datasets presented in this study can be found in online repositories. The names of the repository/repositories and accession number(s) can be found in the article/supplementary material.
